# Differences in host breed and diet influence colonization by *Campylobacter jejuni* and induction of local immune responses in chicken

**DOI:** 10.1186/s13099-016-0133-1

**Published:** 2016-11-10

**Authors:** Zifeng Han, Thomas Willer, Colin Pielsticker, Lenka Gerzova, Ivan Rychlik, Silke Rautenschlein

**Affiliations:** 1 Clinic for Poultry, University of Veterinary Medicine Hannover, Bünteweg 17, 30559 Hannover, Germany; 2 Veterinary Research Institute, Hudcova 70, 621 00 Brno, Czech Republic

**Keywords:** *Campylobacter jejuni*, Breed, Diet, Immune response, Gut microbiota

## Abstract

**Background:**

Chickens are regarded as the main reservoir for human campylobacteriosis. Little is known about the interaction between *Campylobacter jejuni* (*C. jejuni*) and chickens. This interaction may be influenced by the stage of maturation of the immune system, developing gut microbiota composition and other factors including breed and diet. Our aim was to investigate the impact of breed, and diet on *C. jejuni* colonization and host immune responses in chickens. Birds were inoculated with 10^4^ colony forming units (CFU) of *C. jejuni* or diluent at one (Exp. 1) or 22 (Exp. 2) days post hatch. We compared local immune cell subpopulations, cytokine expression levels, and gut microbiota composition between broiler-type (BT) and layer-type (LT) birds fed with either commercial broiler feed (bf) or layer feed (lf).

**Results:**

Lower colonization rates were observed in the older age group independent of breed and diet. Independent of breed, birds fed with bf showed higher CFU of *C. jejuni* compared to lf-fed groups. *Campylobacter jejuni*-inoculation had a significant effect on lymphocyte numbers and cytokine expression levels in BT birds independent of feeding strategy (*p* < 0.05). These effects were not detected in LT birds, only LT birds fed with bf showed a significant increase in IL-8-expression at 7 days post *C. jejuni* inoculation compared to LT-control birds (*p* < 0.05). Diet influenced gut microbiota composition in a comparable manner between BT and LT birds, but changes in microbiota composition associated with *C. jejuni* inoculation varied between breeds.

**Conclusions:**

Diet and breed influenced *C. jejuni* colonization, immune responses and microbiota composition to a different extent comparing between LT and BT birds. The mechanisms behind these differences have to be elucidated further. Our results suggest that selection for more resistant breeds in combination with adapted feeding strategies may help to reduce *Campylobacter* colonization levels in commercial poultry in the future.

## Background

*Campylobacter* species, in particular *Campylobacter jejuni* (*C. jejuni*), cause the majority of human food-borne bacterial gastroenteritis in the industrialized world [1, 2]. *Campylobacter jejuni* is found in a range of domesticated animals, and chickens are the predominant reservoir for *C. jejuni* [3]. So far no suitable strategies have been implemented which allow a reliable prevention of *C. jejuni* colonization of chickens in the field [4]. The reduction of *C. jejuni* colonization rates may be a reachable goal [5, 6]. While pro- and prebiotics have led to inconsistent results [7–9], other control measures including feeding strategies and the use of more resistant breeds may allow significant reduction of *C. jejuni* colonization [10–12].

The induction of local and systemic humoral immune responses [13] have been described after *C. jejuni*-inoculation suggesting that *C. jejuni* may be not only a commensal bacteria of chickens [14]. *Campylobacter jejuni* induced innate immune responses in vitro in different avian cell lines, including HD 11 macrophages, primary chicken kidney cells and primary chicken embryo intestinal cells [15, 16]. In vivo studies demonstrated an increase of proinflammatory cytokines following *C. jejuni* colonization. *Campylobacter jejuni*-inoculated birds showed an increase in the mRNA expression of interleukin (IL)-6 and the chicken IL-8-homolog in ileal and caecal tissues [17]. This may be associated directly with colonizing *Campylobacter* or indirectly with a *C. jejuni* mediated changes in the microbiota composition, including bacterial species such as *Staphylococci*, *Enterococci*, *Enterobactericeae* or *Escherichia coli* [18] and subsequently a modified immune response, which has not become clear so far. However, the immune responses in vivo may be affected and modified by many factors. Most studies are difficult to compare because different *C. jejuni* strains and dosages, different breeds of birds and age groups were used [14, 17, 19–21]. Li et al. demonstrated by caecal transcriptome and gene expression profiling that one broiler line may be more resistant than another line to *C. jejuni* infection [20, 21]. Mainly meat type birds were investigated and different breeds compared [14, 20–22], but field observations also described the disease in layer-type birds [23], which is associated with the sole isolation of *Campylobacter* in affected tissue.

Often only one time point post *C. jejuni* inoculation was investigated not considering the dynamics of colonization [17, 24].

The role of T cells in the control of *C. jejuni* in mice and human beings was demonstrated, but little is known about T cell responses in chickens [15, 25, 26]. It has been suggested that *C. jejuni* infections in avian species are associated with Th1 polarization of the immune response [27, 28].

It can be speculated that changes in the poultry diets may modify the caecal microbiota and gut health of chickens, and therefore pertinently affect the presence of *C. jejuni* in the chicken gut. Recent studies have shown that the feeding strategy can alter the viscosity of gut content as well as the histomorphology of the chicken gut [11], and modify goblet cell glycoconjugates in the intestinal tract in vitro [29]. It is not fully clear how different feeding strategies may alter the colonization pattern of the intestinal bacteria, the development of local immunity and subsequently modulate local immune reactions in response to *C. jejuni* colonization. Most of these studies were conducted in broilers and focused on the relationship between *C. jejuni* colonization and nutritional changes [10, 30–32].

The goal of this study was to investigate the interaction between breed and feeding strategy on *C. jejuni* colonization in commercial hybrid layer and broiler type birds with a cross-over study. *Campylobacter jejuni*-inoculated and non-inoculated groups of both breeds were either fed with commercial broiler-feed (bf) or layer-feed (lf). We investigated local and systemic immune reactions as well as possible differences in gut microbiota composition. Our data clearly demonstrate that feed composition, as well as breed, influenced the outcome of *C. jejuni* colonization, immunity development and the gut microbiota, providing the basis for follow-up studies on the possibility of a reduction of *C. jejuni* colonization by the selection for more resistant breeds in combination with protective feeding strategies.

## Methods

### Animals

Embryonated eggs from commercial layer-type (LT) hybrids (Lohmann Selected Leghorn, LSL) chickens were provided by the KG Geflügelzuchtbetriebe Gudendorf-Ankum GmbH & Co. KG, Ankum, Germany and eggs from the commercial Ross-308 broiler-type (BT) chickens were obtained from the BWE Hatchery Weser-Ems GmbH & Co. KG, Visbek, Rechterfeld, Germany. Eggs were incubated and hatched at the Clinic for Poultry, University of Veterinary Medicine Hannover, Germany. Chickens were housed and raised at the Clinic of Poultry, University of Veterinary Medicine Hannover.

All BT or LT birds were kept in the same room on wood shavings until the age of inoculation. Afterwards, inoculated and non-inoculated experimental groups were moved to different isolation rooms (one for inoculated and one for non-inoculated control birds) with units with wire floors. All groups received feed from the same source (broiler-type or layer-type feed fed to either BT or LT birds).

Commercial broiler and layer feed (Table 1) as well as water were provided ad libitum. Birds were fed a standard starter diet up to 14 days of age and then received a grower diet until the end of the experiment. Birds were distributed randomly to different groups based on SRS (simple random sample), and observed daily for the presence of clinical signs. All birds tested were negative for *C. jejuni* by cloacal swabs on the day of *C. jejuni* inoculation. The animals did not receive any vaccination.

**Table 1:** Ingredients and nutrient contents of the experimental diets

Components, per kg	Broiler-feed		Layer-feed	
	Starter	Grower	Starter	Grower
Crude protein (g)	215	210	180	165
Crude lipids (g)	52	67	38	36
Crude fiber (g)	31	35	47	27
Crude ash (g)	56	51	60	13
MJ ME	12.4	12.4	11.4	11.0
Ca (g)	9	8	10	36
P (g)	6	5.5	6	5
Na (g)	1.4	1.4	1.5	1.6
Methionine (g)	5.5	5.0	3.5	3.5
Lysine (g)	12.5	11.5	8	3.5
Monensin-Na (mg)	100	100	0	0

*MJ ME* megajoule metabolizable energy

### Bacterial strains and *C. jejuni* inoculum preparation

The *C. jejuni* strain of serogroup Lior6 had been isolated from a chicken at the Clinic of Poultry, University of Veterinary Medicine Hannover, Germany and was stored in skim milk at −70 °C [26].

The cryopreserved bacteria were thawed and plated on charcoal cefoperazone dexoxycholate agar (CCDA, Oxoid, Basingstoke, England). The plates were incubated for 48 h under microaerophilic conditions (10% CO_2_, 5% O_2_, 85% hydrogen) at 38 °C. After 2 days, one *C. jejuni* colony was transferred into 3 ml Standard-I-Bouillon (Merck, Damstadt, Germany) and incubated for another 48 h under microaerophilic conditions at 38 °C.

One milliliter of the bacterial suspension was diluted with sterile phosphate buffered saline (PBS) to achieve approximately 10^4^ CFU/ml (Colony Forming Units) for oral inoculation. To confirm the CFU of *C. jejuni* in the inocula, the bacterial suspension was serially diluted in a 10-fold dilution series, spread on CCDA plates and incubated for 48 h at 38 °C. After incubation the colonies were counted to calculate the CFU [17].

### Isolation of intraepithelial lymphocytes and flow cytometric analysis

Single cell suspensions of intraepithelial lymphocytes (IEL) were prepared as previously described in detail [33].

10^6^ IEL of the caecum were triplestained with a combination of the following antibodies (final concentrations per ml): mouse-anti-chicken-CD3 (2 µg) [34] conjugated to phycoerythrin (R-PE), biotinylated mouse-anti-chicken-CD8β (5 µg) [35] used in conjunction with streptavidin conjugated to SpectralRed™ (SPRD) (5 µg) and fluorescein (FITC)-conjugated mouse-anti-chicken CD4 (5 µg). All antibodies were obtained from Southern Biotech, provided by Biozol, Eching, Germany. The lymphocyte population was gated for CD3+ IELs according to size and granularity and 200,000 events per caecum sample were measured for R-PE, FITC or SPRD positive staining with a Beckman Coulter Epics XL*©* flow cytometer. The stained cells were analyzed by using the EXPO 32 ADC software program (Beckman Coulter Company, Miami, FL). CD3+ IELs were then analyzed for positive staining with anti-CD4-FITC and anti-CD8-SPRD. Presented is the percentage of CD4+ and CD8+ T lymphocytes within the CD3+ cell population.

### Histology

Samples of liver and middle-caecum were collected, fixed in phosphate-buffered formalin (4%) for 24 h and further processed for histological examination following standard procedures. The different tissue sections of 2 µm were investigated microscopically for histopathological lesions such as oedema in the lamina propria of caecum or crypt abscesses and cell ballooning as previously described in mice and chicken after *C. jejuni* inoculation [36, 37].

### Immunohistochemistry

Frozen sections of middle caecum were processed as previously described [33, 38]. Sections were stained with one of the following mouse-anti-chicken unlabeled monoclonal antibodies: anti-CD4, anti-CD8β and anti-Bu1 (0.05 µg/ml) (Southern Biotech, provided by Biozol, Eching, Germany). The secondary anti-mouse IgG biotinylated antibodies and ABC reagent (Vectastain^®^ Elite^®^ ABC Kit, Vector Laboratories Inc., provided by Linaris, Wertheim-Bettingen, Germany) were applied according to the manufacturer’s instructions. The enzyme-linked ABC complex was visualized by the reaction with 3.3′-diaminobenzidine (DAB) chromagen substrate and hydrogen peroxide (DAB peroxidase substrate Kit, Vector Laboratories Inc.). Sections were examined with light microscopy. The different lymphocyte populations were evaluated by counting the number of positive stained cells per 3 crypts in the lamina propria of 5 representative microscopic fields at 200× optical magnification per animal [33].

### Real-time quantitative RT-PCR (qRT-PCR)

Total RNA was isolated from caecum samples with 1000 µl Trifast^®^-GOLD reagent (PeqLab, Biotechnologie GmbH, Erlangen, Germany) according to the manufacturer’s instructions. RNA-quality and concentrations were determined using the NanoDrop ND-1000 (PeqLab, Biotechnologie GmbH).

All details of the specific primers and probes for the detection of expressed cytokines IL-6 and the chicken IL-8 homolog as well as the house-keeping gene 28S have been described previously [15, 39]. Real-time quantitative RT-PCR was performed using the AgPath-ID One-Step RT-PCR Kit (Applied Biosystems, Ambion, USA). Amplification and quantification of the specific products was carried out by using the Mx3005P™ thermal cycle system and Mx3005P™ Q PCR Software (STRATAGENE, Agilent Technologies Company, USA), respectively. The following cycle profile was applied: one cycle at 50 °C for 30 min and 95 °C for 10 min, and 40 cycles at 95 °C for 20 s and 60 °C for 1 min.

The results were normalized with the house-keeping gene 28S [40], the expression of which was comparable between birds of *C. jejuni*-inoculated and non-inoculated ones, and were expressed as 40-Ct in mRNA expression in the tissues of *C. jejuni* inoculated birds and *C. jejuni*-free controls.

### DNA purification and pyrosequencing

Microbiota was characterized by the next generation sequencing of the V3/V4 variable region of 16S rRNA genes. Caecal samples were homogenized using zirconia silica beads (BioSpec Products) in a Mag NALyzer (Roche Diagnostics). Following homogenization, the DNA was extracted using the QIAamp DNA Stool Mini Kit according to the manufacturer’s instructions (Qiagen). The DNA concentration and quality was determined spectrophotometrically and the DNA was stored at −20 °C until use. Prior to PCR, DNA samples were diluted to 5 ng/μl and used as a template with forward primer 5′-TCGTCGGCAGCGTCAGATGTGTATAAGAGACAG -MID- *GT*-*CCTACGGGNGGCWGCAG*-3′ and reverse primer 5′-GTCTCGTGGGCTCGGAGATGTGTATAAGAGACAG -MID- *GT*-*GACTACHVGGGTATCTAATCC*-3′. The sequences in italics served as an index and adapter ligation while underlined sequences allowed an amplification over the V3/V4 region of 16S rRNA genes. MIDs represent different sequences of 5, 6, 9 or 12 bp in length designed to differentiate samples. PCR amplification and clean- up were performed using KAPA Taq HotStart PCR kit (Kapa Biosystems). In the next step the DNA concentration was determined fluorometrically and the DNA was diluted to 100 ng/µl. Groups of 14 PCR products with the same MID sequences were indexed with a Nextera XT Index Kit following the manufacturer’s instructions (Illumina). Prior to sequencing, the concentration of differently indexed samples was determined using a KAPA Library Quantification Complete kit (Kapa Biosystems). All indexed samples were diluted to 4 ng/µl and 20% of phiX DNA was added. Sequencing was performed using MiSeq Reagent Kit v3 and MiSEQ 2000 apparatus according to the manufacturer’s instructions (Illumina).

### Sequence analysis

Fasta and qual files generated after Illumina sequencing were uploaded into Qiime software [41]. Reverse reads were shortened to a length of 250 bp and forward and reverse sequences were joined. Quality trimming criteria were set to a value of 19 and no mismatch in the MID sequences. In the next step, chimeric sequences were predicted by slayer algorithm and excluded from subsequent analysis. The resulting sequences were then classified by RDP Seqmatch with an OTU (operational taxonomic units) discrimination level set to 97% followed by UniFrac analysis [42]. Principal coordinate analysis (PCoA) was used for data visualization.

### Experimental design

#### Experiment 1 (Exp. 1)

72 commercial broilers and 72 commercial layer pullets were divided into two subgroups. Subgroups of *C. jejuni*-free commercial broilers and layer pullets were fed either with broiler feed (bf) or layer feed (lf). 18 birds per subgroup were orally inoculated with *C. jejuni* strain Lior 6 at 1 day post hatch (dph) by crop inoculation with a dose of approximately 10^4^ colony-forming units (CFU) or *C. jejuni*-free medium. Six birds of each subgroup were randomly selected at a specific point of time and necropsied at 1, 7 and 14 days post inoculation (dpi). Individual body weight and pathological lesions were determined. To avoid cross contamination, liver samples were collected under sterile conditions at the first step of necropsy, and subsequently were investigated for *C. jejuni* by taking direct swabs from the depth of the parenchym. Additionally, caecal content was analyzed for the number of CFU of *C. jejuni/*g caecal content. Samples of middle caecum were taken for immunohistochemical staining of local lamina propria lymphocyte (LPL) populations. IELs of caecum were isolated for the flow cytometric analysis of T cell subpopulations. Middle caecum from birds was also evaluated for cytokine expression levels at 1 and 7 dpi by using quantitative real-time RT-PCR (qRT-PCR). In addition, the caecal content of birds was collected at 7 dpi and investigated for gut microbiota composition.

#### Experiment 2 (Exp. 2)

Exp. 1 was repeated with 22-days-old birds. 72 commercial broilers and 72 commercial layer pullets were divided into two subgroups. 18 birds per subgroup were orally inoculated with either *C. jejuni*-free medium or approximately 10^4^ CFU of *C. jejuni* at 22 dph. Most parameters were investigated as described in Exp. 1. Cytokine expression levels and gut microbiota composition were not determined in this experiment.

### Statistical analysis

Statistical analyses were carried out with the Statistix version 10.0 (Analytical software, Thallahassee, FL, USA). For the statistical analysis of differences in the CFU numbers of *C. jejuni* of different *C. jejuni*-inoculated subgroups of the same age at the indicated dpi, Kruskal–Wallis all-pairwise comparisons test was used. The differences in the IEL T cell subsets and the number of LPL immune cell populations between *C. jejuni*-inoculated and non-inoculated controls were determined by Two-Sample *T* test or Wilcoxon Rank sum *T* test, respectively. The difference in cytokine expression level between *C. jejuni*-inoculated and *C. jejuni*-free control groups was determined by Two-Sample *T* test. Statistical significance was designated as *p* < 0.05.

## Results

### Clinical signs and tissue lesions

No clinical signs, macroscopical or microscopical lesions were observed in the caecum of either *C. jejuni*-free control or *C. jejuni*-inoculated groups in both experiments. No significant differences in body weight were detected between *C. jejuni*-inoculated and non-inoculated control birds within each breed and diet-group at the different necropsy days (data not shown, *p* > 0.05). BT and LT birds fed with layer-feed (lf) had significantly lower body weight on all necropsy days compared to broiler-feed (bf) fed groups of the respective breed (data not shown, *p* < 0.05).

### Qualitative detection of *C. jejuni* in liver

*Campylobacter jejuni* positive-liver samples were only observed in LT-bf birds, which had been inoculated with *C. jejuni* at 1 dph, with one of six and two of six chickens having a *C. jejuni* positive liver at 1 and 7 dpi respectively.

### Quantitative detection of *C. jejuni* in caecal content

Caecal content was evaluated for *C. jejuni* quantitatively at 1, 7 and 14 dpi. Lower CFU numbers as well as a lower colonization rate were observed in birds, which had been inoculated at 22 dph compared to birds inoculated at 1 dph at all investigated time points. Feed and breed influenced the colonization pattern of *C. jejuni*. LT birds fed with bf, which had been inoculated at 1 dph, showed significantly higher numbers of CFU of *C. jejuni* compared to LT birds fed with lf at 1, 7 and 14 dpi indicating the effect of feed (Table 2, *p* < 0.05). This observation was confirmed also with LT birds, which had been inoculated at 22 dph, at 1 and 7 dpi, but the difference was not significant due to higher individual variation.

**Table 2:** Average number of colony forming units of *C. jejuni* in caecal content of broiler- and layer-type birds fed with either broiler- or layer-feed

Breed-feed	Inoculation day (dph)	Average number of CFU of *C. jejuni* [range] in caecal content of birds at dpi (log_10_ CFU/g)			Colonizatioin rate at each dpi in % *C. jejuni* positive birds of total inoculated birds		
		1	7	14	1	7	14
BT-bf	1	7.07^ab^ [6.53–7.38]	7.91^b^ [7.63–8.63]	8.10^b^ [7.89–8.27]	100	100	100
BT-lf	1	7.15^ab^ [5.72–7.50]	7.69^b^ [7.30–8.36]	7.66^b^ [6.70–8.12]	100	100	100
LT-bf	1	7.58^a^ [6.87–8.48]	8.84^a^ [8.22–9.19]	8.85^a^ [8.62–9.01]	100	100	100
LT-lf	1	6.51^b^ [5.66–7.37]	7.09^b^ [5.71–8.06]	8.01^b^ [7.28–8.54]	100	100	100
BT-bf	22	2.51^a^ [0.00–6.13]	6.77^b^ [4.81–7.98]	4.41^ab^ [0.00–6.65]	50	100	83.3
BT-lf	22	1.51^a^ [0.00–4.82]	6.01^ab^ [5.26–7.05]	5.70^b^ [3.77–7.10]	33.3	100	100
LT-bf	22	2.65^a^ [0.00–5.86]	5.94^ab^ [4.50–7.38]	1.33^a^ [0.00–4.42]	50	100	33.3
LT-lf	22	0.00^a^ [0.00–0.00]	3.89^a^ [0.00–6.03]	5.02^b^ [0.00–6.21]	0	83.3	83.3

BT and LT, which were fed with either bf or lf, were orally inoculated with approximately 10^4^ CFU of *C. jejuni* at 1 (Experiment 1) or 22 (Experiment 2) dph

*BT* broiler-type birds and* LT* layer-type bird fed with either* bf*  broiler feed or* lf* layer feed, *CFU* colony forming units, *dpi* days post inoculation, *dph* days post hatch

^ab^Different superscript letters indicate significant differences between groups of the same age at the indicated days post *C. jejuni* inoculation (n = 6/group, *p* < 0.05)

In addition in Exp. 1, LT birds fed with bf showed higher CFU numbers of *C. jejuni* compared to BT birds on most of the investigated points of time. This effect was only detected in Exp. 2 at 1 dpi, while at later points of time BT birds had higher colonization rates than LT birds.

### Detection of local intestinal lymphocyte populations

#### Immunohistochemical detection of T and B lymphocytes in the lamina propria of caecum

**Fig. 1:**
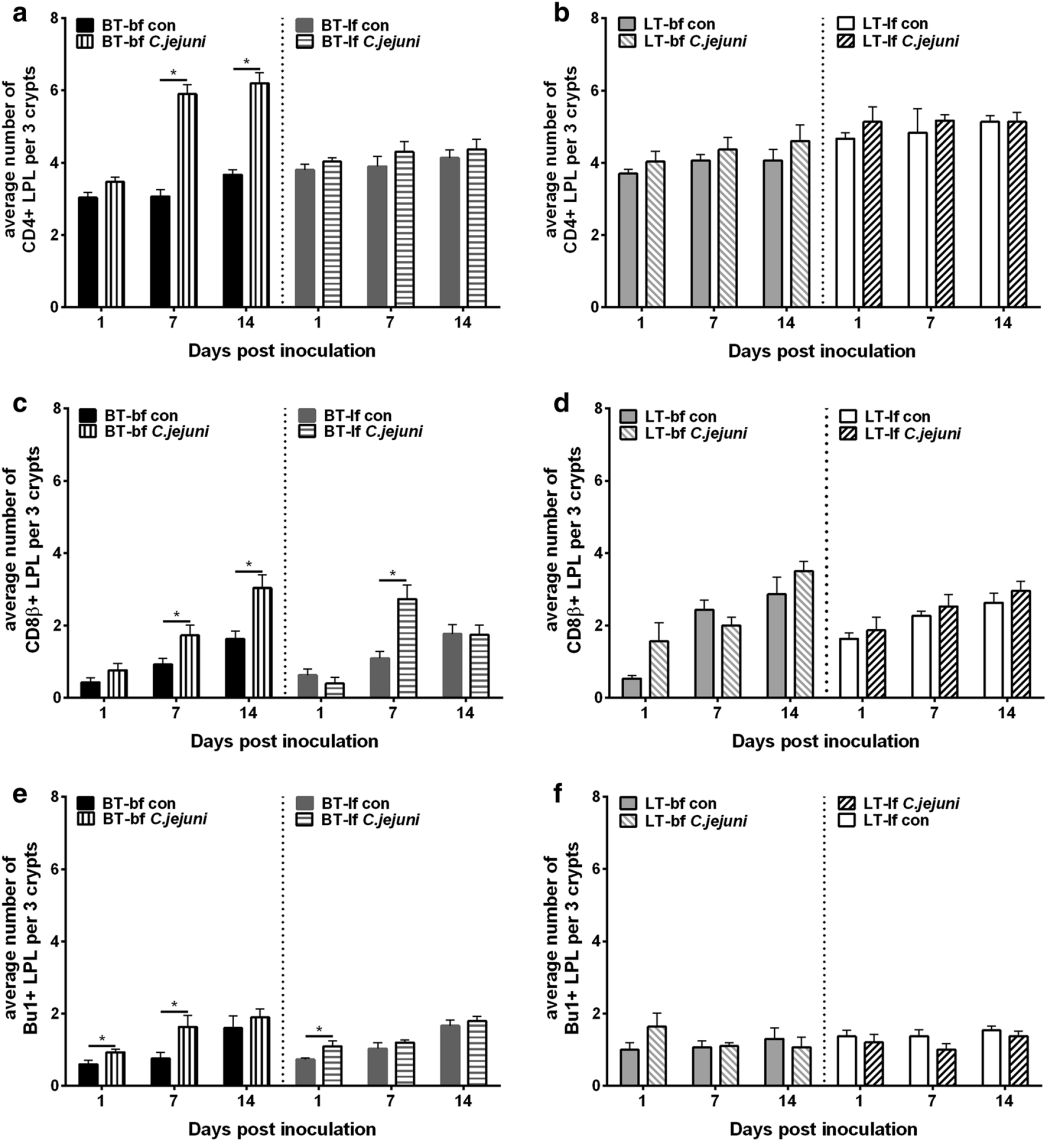
Immunohistochemical detection of T and B lymphocytes in caecum of 1-day-old birds. Immunohistochemical detection of CD4+ (**a**, **b**), CD8β+ (**c**, **d**) and Bu1+ (**e**, **f**) lymphocytes in caecal lamina propria of birds, which had been inoculated with either *C. jejuni* or *C. jejuni*-free medium at 1 day post hatch (Experiment 1). Broiler-type (BT) (**a**, **c**, **e**) and layer-type (LT) (**b**, **d**, **f**) birds, which were fed with either broiler feed (bf) or layer feed (lf). *Asterisk* letters indicate significant differences between *C. jejuni*-inoculated (*C. jejuni*) and *C. jejuni*-free control (con) groups at the indicated days post *C. jejuni* inoculation (n = 6/group, *p* < 0.05)

**Fig. 2:**
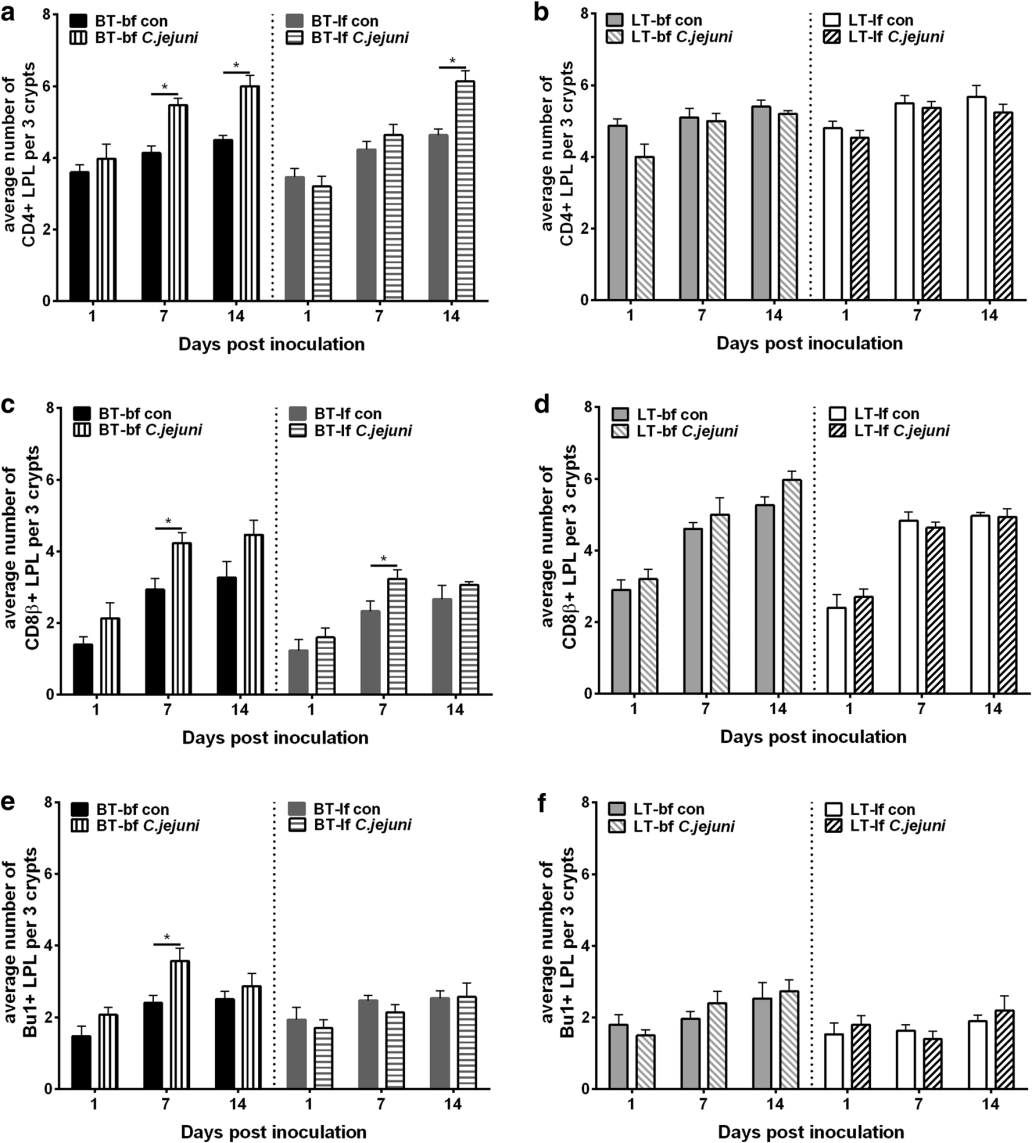
Immunohistochemical detection of T and B lymphocytes in caecum of 22-days-old birds. Immunohistochemical detection of CD4+ (**a**, **b**), CD8β+ (**c**, **d**) and Bu1+ (**e**, **f**) lymphocytes in caecal lamina propria of birds, which had been inoculated with either *C. jejuni* or *C. jejuni*-free medium at 22 day post hatch (Experiment 2). Broiler-type (BT) (**a**, **c**, **e**) and layer-type (LT) (**b**, **d**, **f**) birds, which were fed with either broiler feed (bf) or layer feed (lf). *Asterisk* letters indicate significant differences between *C. jejuni*-inoculated (*C. jejuni*) and *C. jejuni*-free control (con) groups at the indicated days post *C. jejuni* inoculation (n = 6/group, *p* < 0.05)

As expected based on previous studies [17], the numbers of T- and B-lymphocyte populations in the lamina propria were influenced by age. Caecal CD4+ , CD8+ and Bu1+ LPL gradually increased in control birds over time (Figs. 1, 2).

Breed influenced the number of T- and B-lymphocyte populations in LPL. Independent of feeding strategy and age, higher numbers of caecal CD4+ and CD8+ cells were observed in *C. jejuni*-free LT birds compared to *C. jejuni*-free BT birds (Figs. 1, 2).

A significant increase in B lymphocytes as well as CD4+ and CD8+ T lymphocytes in caecal lamina propria was observed after *C. jejuni* inoculation of 1-day-old as well as 22-days-old BT but not in LT chickens. *Campylobacter jejuni*-inoculated BT-bf birds showed an increase in the number of caecal CD4+ and CD8+ T cells at 7 and 14 dpi (Figs. 1, 2). B lymphocytes increased at 1 and 7 dpi, which was significant for the younger age group (*p* < 0.05). Fewer changes in lymphocyte numbers were observed in BT-lf chickens in which a significant increase was recorded only in CD8+ T lymphocytes at 7 dpi for both age groups and B lymphocytes at 1 dpi for the younger age group (Figs. 1, 2). In LT birds, a transient increase in numbers of CD8+ T lymphocytes and B lymphocytes was observed also in the caecum of LT-bf chickens at 1 dpi (*p* > 0.05).

Overall, there was a significant effect of *C. jejuni* colonization and breed on LPL populations in caecum (*p* < *0.05*).

### Detection of IEL in caecum

**Fig. 3:**
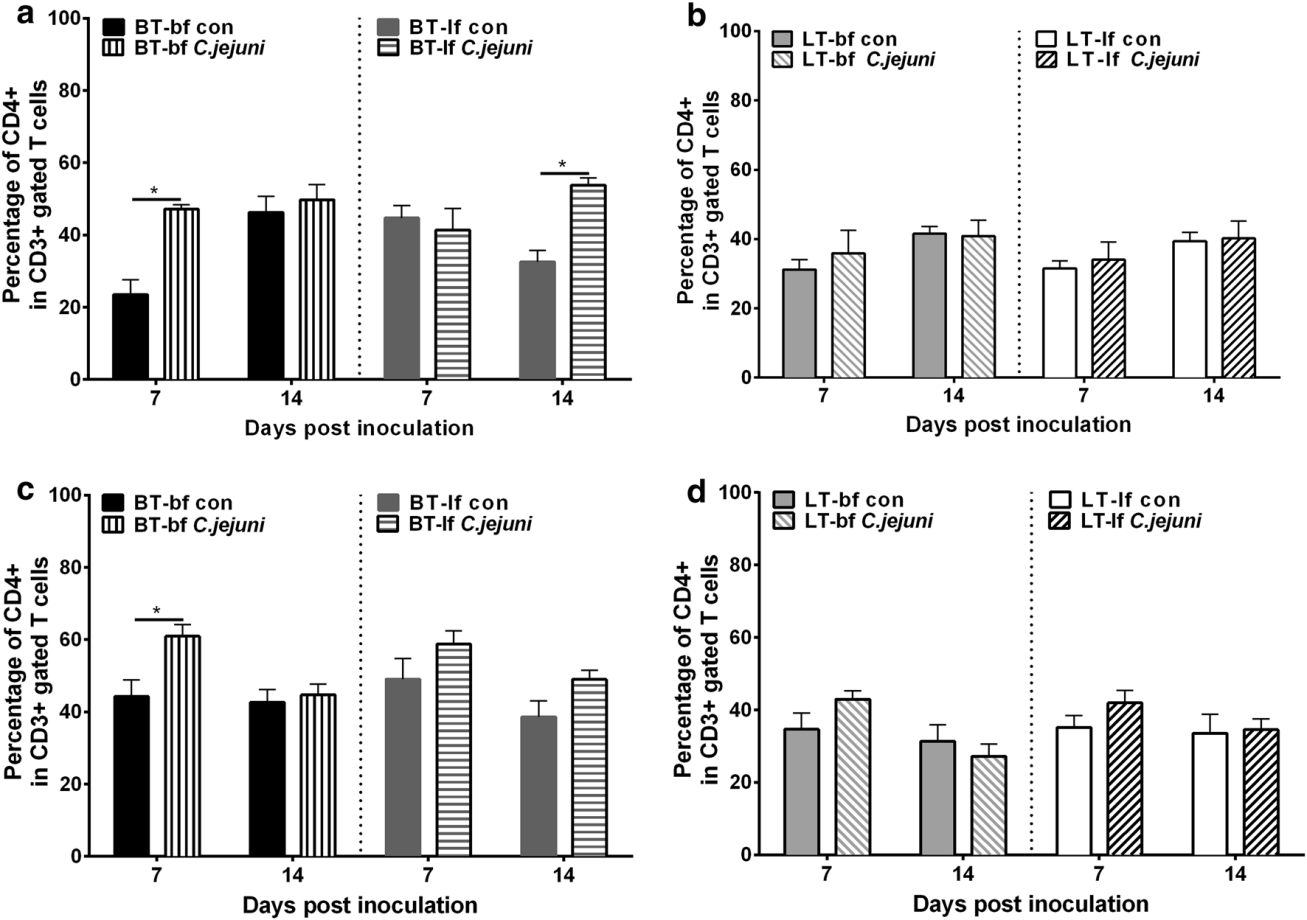
Flow cytometric analysis of T lymphocytes. Flow cytometric analysis of the percentage of CD4+ intraepithelial lymphocytes (IEL) in caecum of broiler-type (BT) (**a**, **c**) and layer-type (LT) birds (**b**, **d**) fed with either broiler feed (bf) or layer feed (lf). Birds had been *C. jejuni*-inoculated at 1 (Exp. 1) (**a**, **b**) and 22 (Exp. 2) (**c**, **d**) days post hatch. CD4+ T cells were gated within the CD3+ IEL population. *Asterisk* letters indicate significant differences between *C. jejuni*-inoculated (*C. jejuni*) and *C. jejuni*-free control (con) groups at the indicated days post *C. jejuni* inoculation (n = 6/group, *p* < 0.05)

*Campylobacter jejuni* inoculation affected IEL cell numbers only in BT birds, but not in LT chickens, indicating a breed effect (Fig. 3) (*p* < 0.05). A significant upregulation of CD4+ intraepithelial T cells (Fig. 3a, c) was observed in *C. jejuni*-inoculated BT-bf birds at 7 dpi for both age groups compared to *C. jejuni*-free control groups (*p* < 0.05). *Campylobacter jejuni*-inoculated BT-lf birds showed up-regulation in CD4+ IEL numbers only at 14 dpi in birds, which had been inoculated at 1 dph (Fig. 3a). Numbers of CD8+ IEL were not affected in either experiment.

### Detection of cytokine mRNA expression levels

For this investigation we selected birds, which had been inoculated at 1 dph and focused on 1 and 7 dpi, because most prominent changes in immune cell numbers were observed in this age group [15, 43].

**Fig. 4:**
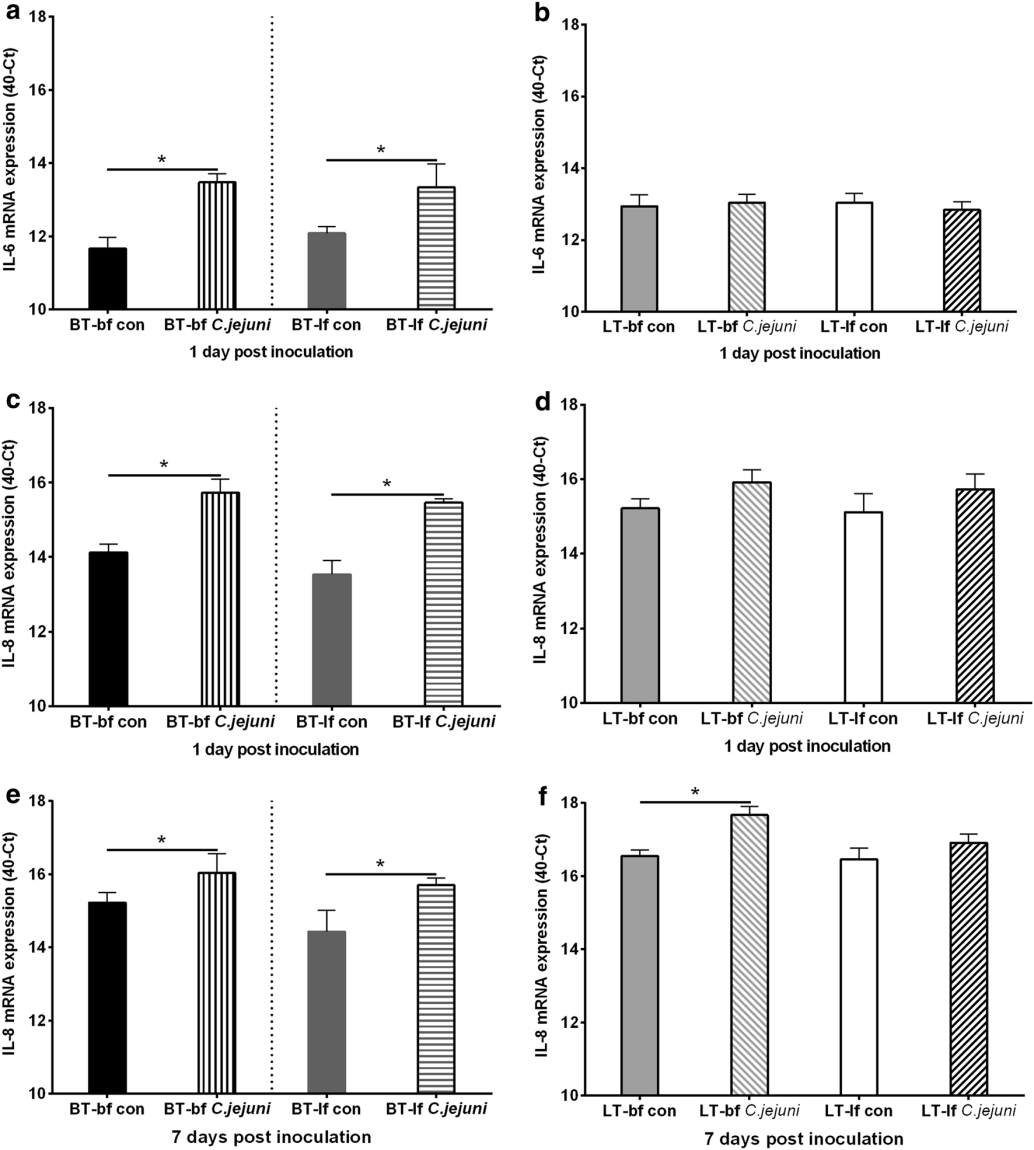
mRNA expression levels in caecum of 1-day-old birds. IL-6 (**a**, **b**) and IL-8 (**c**–**f**) mRNA expression levels in caecum samples of broiler-type (BT; **a**, **c**, **e**) and layer-type (LT; **b**, **d**, **f**) birds fed with either broiler feed (bf) or layer feed (lf). Birds had been *C. jejuni*-inoculated at 1 day post hatch. Comparison of cytokine mRNA expression between *C. jejuni*-free control and *C. jejuni*-inoculated birds at 1 dpi (**a**–**d**). Comparison of cytokine mRNA expression between *C. jejuni*-free control and *C. jejuni*-inoculated birds at 7 dpi (**e**, **f**). Data are presented as the mean mRNA expression (40-Ct) normalized to 28S. *Asterisk* letters indicate significant differences between *C. jejuni*-inoculated (*C. jejuni*) and *C. jejuni*-free control (con) groups at the indicated days post *C. jejuni* inoculation (n = 6/group, *p* < 0.05)

We observed a breed and *C. jejuni* effect. Independent of *C. jejuni*-infection and feeding strategy, *C. jejuni*-free layers showed significant higher IL-6 mRNA expression levels only at 1 dpi, as well as IL-8 mRNA expression levels at both 1 and 7 dpi compared to *C. jejuni*-free broilers (Fig. 4), indicating a breed effect (*p* < *0.05*).

*Campylobacter jejuni* colonization modified the expression level of the chicken IL-8-homolog and IL-6 significantly in BT birds (*p* < 0.05). Independent of feeding strategy, significant up-regulations were detected in *C. jejuni*-inoculated BT birds for both IL-6 and IL-8 at 1 dpi (Fig. 4a, c) as well as for IL-8 at 7 dpi (Fig. 4e) compared to *C. jejuni*-free groups (*p* < 0.05). A significant up-regulation of IL-8 was also observed at 7 dpi in the caecum of *C. jejuni*-inoculated LT-bf birds. IL-6 mRNA expression levels were not significantly different between *C. jejuni*-inoculated and non-inoculated groups in both BT and LT birds at 7 dpi (data not shown).

### Gut microbiota composition

**Fig. 5:**
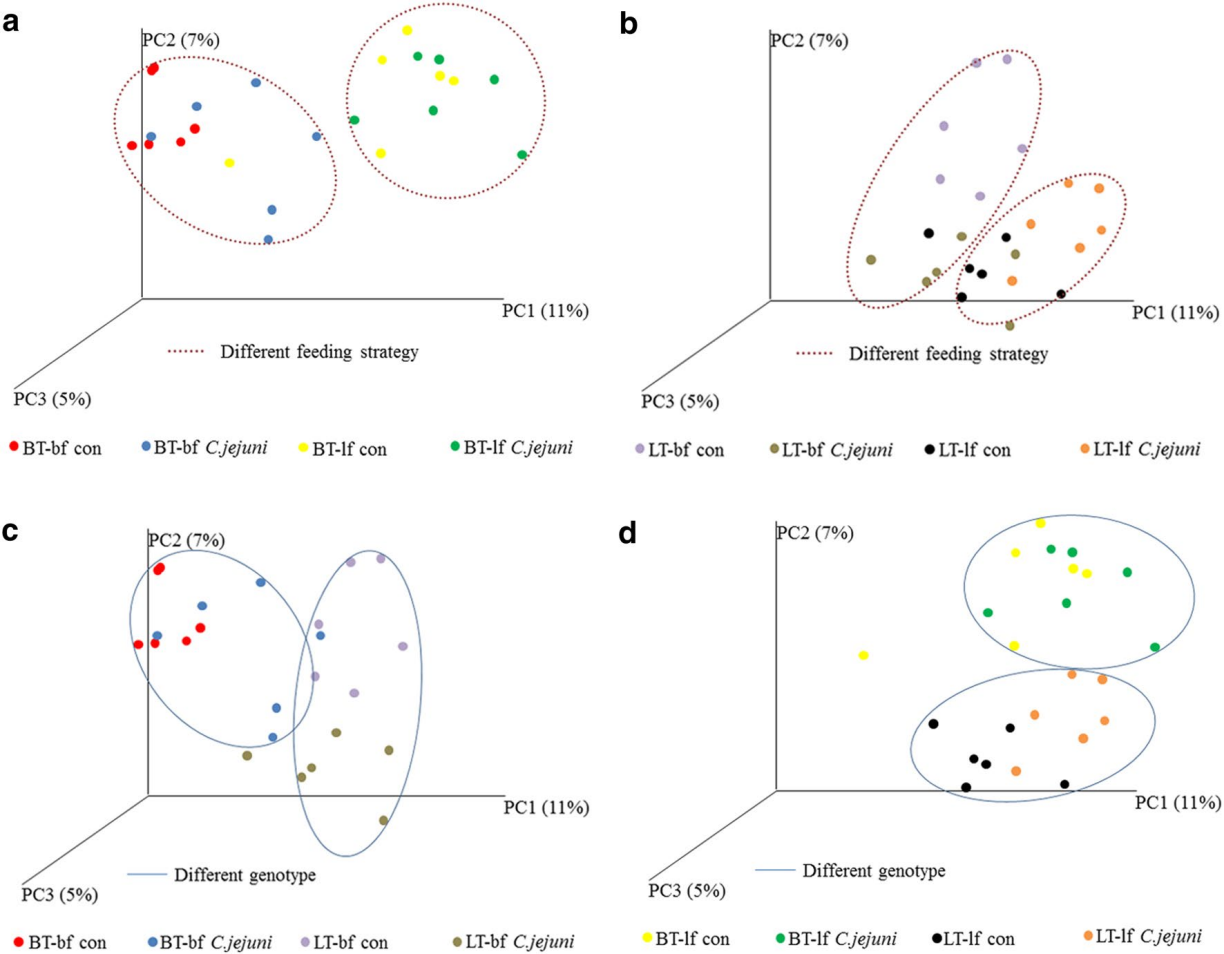
Microbiota diversity. Microbiota diversity in caecum of broiler-type (BT) and layer-type (LT) birds fed with either broiler feed (bf) or layer feed (lf). UniFrac analysis followed by PCoA indicates variability in the caecal microbiota composition based on different feeding strategy (**a**, **b**) and genetic background (**c**, **d**). “con” = *C. jejuni*-free control, “*C. jejuni*” = *C. jejuni*-inoculated. (Figures were generated from raw data but when we produced the figures from normalized data, these were essentially the same. We therefore used maximal data available for each sample in this figure)

**Fig. 6:**
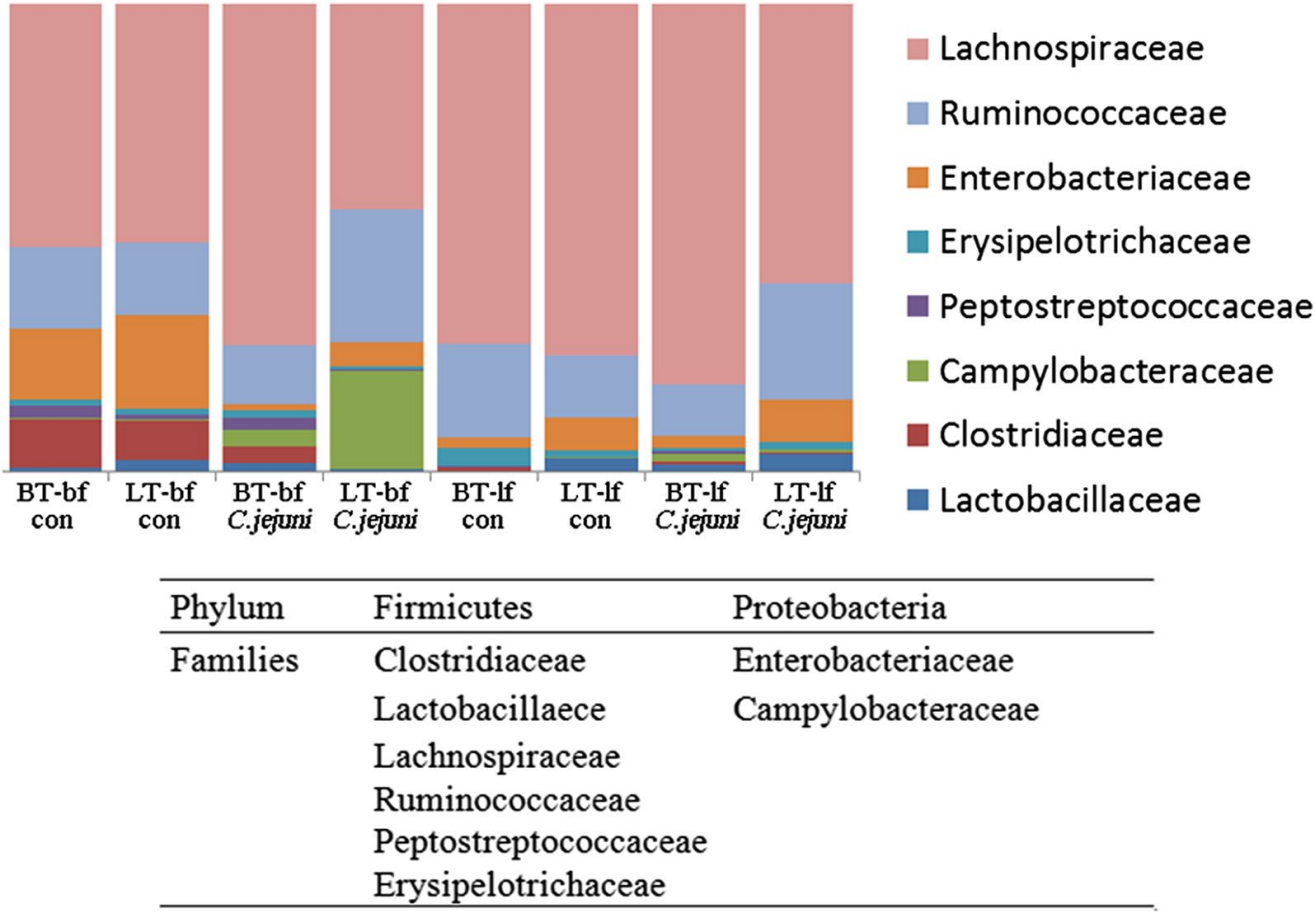
Gut microbiota composition. The composition of the main families present caecal microbiota in chicken. The sum of the appropriate families as indicated in the figure legend provides information on the microbiota distribution at the phylum level. Taxonomy summary and microbial diversity of the operational taxonomic units (OTU) from caecal samples collected at 7 days post inoculation from broiler-type (BT) and layer-type (LT) birds fed with either broiler feed (bf) or layer feed (lf), which had been *C. jejuni*-inoculated at 1 day post hatch (n = 6 per group). “con” = *C. jejuni*-free control, “*C. jejuni*” = *C. jejuni*-inoculation. (Figures were generated from raw data but when we produced the figures from normalized data, these were essentially the same. We therefore used maximal data available for each sample in this figure)

UniFrac analysis followed by PCoA indicated effects of genetic background and feeding strategy using both unweighted and weighted analyses since clear separations were observed between subgroups (Fig. 5). This analysis also indicated a difference in gut microbiota composition between *C. jejuni*-inoculated and control groups (Fig. 5). Non-inoculated broilers fed with bf diet had a higher abundance of *Enterobacteriaceae* and *Clostridiaceae*, and a lower abundance of *Lachnospiraceae*, in comparison to the non-infected birds fed with lf diet. In the absence of *C. jejuni* infection, chicken breed had a less significant impact on microbiota composition than feed composition (Fig. 6). After the *C. jejuni* inoculation, more significant differences in microbiota composition were observed in chickens fed bf than in chickens provided with lf diet. Irrespective of diet, microbiota composition of LT chickens changed to a greater extent after *C. jejuni* inoculation than the microbiota of BT chickens (Fig. 6).

## Discussion

No studies have been conducted so far that combined a comparison of genetically different chickens and different diets. We selected two commercially commonly applied breeds of broiler- and layer-type birds to be as close as possible to the field situation. Both were fed with commercially available broiler or layer feed in each combination. Two age groups of 1 (Exp. 1) and 22 (Exp. 2) days old chicken were inoculated with *C. jejuni* and investigated for *C. jejuni*-colonization pattern, local T and B cell numbers, and for the younger age group for IL-6 as well as IL-8-homolog mRNA expression in caecum and gut microbiota composition. Consistent with previous investigations [27, 36, 44], none of the *C. jejuni*-inoculated subgroups showed any clinical signs, pathological or histopathological lesions. We observed different immune reactions and colonization patterns between BT and LT birds, age groups, as well as different feeding groups.

Breed has been considered to be one of the key factors influencing the host response and outcome of *C. jejuni* colonization [14, 20]. To the best of our knowledge, no investigations were conducted to demonstrate breed effects between BT and LT birds. Other studies compared mainly between different broiler lines [14, 20, 21]. Some broiler lines were shown to be more susceptible to *C. jejuni* infection, which were characterized by diarrhea, a prolonged inflammatory response and induction of lymphocyte activation, compared to less susceptible breeds [14, 20]. The mechanisms behind these differences are not known. Possible modification of breed differences by feed composition has not been investigated before.

In our experiment we observed differences in host immune responses between BT and LT chickens. Comparing *C. jejuni*-free groups, LT birds showed higher numbers of caecal CD4+ and CD8+ LPL than BT chickens independent of the investigated age. mRNA expression levels of IL-6 and IL-8 were also higher in the caecum of non-inoculated LT chickens, compared to *C. jejuni*-free broilers independent of feeding strategy and microbiota composition (Exp. 1). It was demonstrated before that the breed may significantly affect early cytokine mRNA expression in the caecum of 1-day-old chickens with and without *Salmonella enteritidis* infection [45]. These differences in immune cell numbers and cytokine expression pattern in *C. jejuni*-free BT and LT birds may explain why also differences in the immune response were detected after *C. jejuni* inoculation of these different breeds. We observed that *C. jejuni*-inoculated broilers showed an upregulation of CD4+ and CD8+ T cells in the lamina propria independent of feeding strategy, which was significant for CD4+ T cells for both age groups compared to *C. jejuni*-free controls (*p* < 0.05). While a clear upregulation of immune cell numbers and cytokine expression was observed in *C. jejuni*-inoculated BT birds, *C. jejuni*-inoculated LT birds did not show any significant changes, which suggests that *C. jejuni*-related changes in microbiota composition alone are not sufficient to modify cytokine expression pattern. Differences in the magnitude of immune responses between LT- and BT-birds were also detected after the infection with other avian pathogens such as *Salmonella* spp., infectious bursal disease virus, or Marek’s disease virus [46–50].

To further understand the role of cellular immunity in *C. jejuni* infection in chicken, we investigated the mRNA expression level of IL-6 and IL-8. It is evident that IL-6 plays an important role in the maintenance of the intestinal epithelium and also in transiting innate to the adaptive immunity [51]. IL-8 is a potent chemokine and an inducer of local inflammatory responses, which attracts and activates macrophages and leukocytes [52, 53]. It has been reported that IL-8 can contribute to the clearance of *C. jejuni* by inducing the activation of neutrophils and T cell subpopulations [54]. Consistent with previous studies [14, 17], mRNA expression levels of IL-6 and IL-8 were increased in *C. jejuni*-inoculated birds compared to *C. jejuni*-free controls. The difference was significant for both cytokines in BT at 1 and 7 dpi independent of diet, but only for IL-8 in LT-bf birds at 7 dpi, indicating that BT birds might mount a more vigorous immune response after *C. jejuni* inoculation compared to LT chickens. The difference in background expression levels between BT and LT may contribute to this observation.

Changing poultry diet may modify the caecal microbiota and gut health of chicken, and therefore may affect the colonization pattern of *C. jejuni* [11, 12]. Recent studies have shown that maize- or wheat-based diets, which contain different levels of crude protein, can alter the viscosity of gut content and histomorphology of the chicken gut [11], and subsequently reduce *C. jejuni* colonization in broilers [9]. Changes in diet due to different protein sources or non-antibiotic feed additive may modify the gastrointestinal environment creating disturbances in the resident microbiota thus allowing—directly or indirectly—either a proliferation or reduction of bacterial pathogens [10, 55]. We observed that LT-bf birds, which were inoculated with *C. jejuni* at 1 dph, showed a significantly higher numbers of CFU of *C. jejuni* compared to LT-lf chickens (*p* < 0.05). Crude protein and fat levels in broiler feed were higher than in layer feed in both starter and grower diet. Other studies have shown that the numbers of caecal CFU of *C. jejuni* were significantly lower in birds fed with protein derived from plants compared to groups which received animal-protein-based feed [56]. Evidence for the role of protein and fat in *C. jejuni* colonization is also given from investigations of mice [57]. Mice were fed with either murine diet or “human cafeteria diet” such as curry sausages, fried chicken nuggets, French fries, and others, which have a higher level of protein and fat. Obesity-induced mice were more susceptible to *C. jejuni* infection compared to the standard diet group [57]. This observation was also confirmed in dogs [58]. This diet influence however, was not significant between the two BT subgroups possibly due to the more vigorous immune response compensating feed effects.

In poultry, it was demonstrated that higher fiber levels in diet may enhance short chain fatty acid production, gut metabolism, and intestinal immunity [59, 60]. We observed a higher fiber content in the starter layer diet with 47% compared to 31% in broiler feed, which may also have contributed to the differences observed between feeding groups.

Different *C. jejuni* strains may have different potential for systemic invasion [61]. On the other side host factors, feed or other so far undefined factors may contribute to the evasion of *C. jejuni* from the gut. *C. jejuni* strain Lior6 was not detected in the liver of BT and LT birds in previous studies [26]. Interestingly, in this study *C. jejuni* invaded the liver of LT-bf birds which also showed the highest level of CFU of *C. jejuni* in the caecum. It may be speculated that there may be a correlation between CFU in the gut and intestinal permeability allowing evasion of *Campylobacter* to other tissues [62].

Overall, our results provide circumstantial evidence that the colonization pattern of commensal bacteria and development of local immunity may be influenced by the breed. Independent of feeding strategy, BT chickens mounted a more vigorous immune response compared to LT birds following *C. jejuni* inoculation. Feeding strategy affected the caecal microbiota composition of both BT and LT birds, and significantly influenced the outcome of *C. jejuni* colonization in LT birds. Further studies should be carried out to understand which components of gut microbiota may influence local immune development and the outcome of *C. jejuni* infection. This should be investigated by considering possible breed differences and may open up new strategies to reduce *C. jejuni* on the poultry flock level and subsequently may reduce the risk of human infections.

## Abbreviations

*C. jejuni*: *Campylobacter jejuni*
BT: broiler-type
LT: layer-type
bf: broiler feed
lf: layer feed
CFU: colony forming units
dph: days post hatch
IL: interleukin
CCDA: charcoal cefoperazone dexoxycholate agar
PBS: phosphate buffered saline
IEL: intraepithelial lymphocyte
OTU: operational taxonomic units
PCoA: principal coordinate analysis
dpi: days post inoculation
LPL: lamina propria lymphocyte
